# Evolution of a novel cell type in Dictyostelia required gene duplication of a cudA-like transcription factor

**DOI:** 10.1016/j.cub.2021.11.047

**Published:** 2022-01-24

**Authors:** Koryu Kin, Zhi-Hui Chen, Gillian Forbes, Pauline Schaap

**Affiliations:** 1 University of Dundee, School of Life Sciences, Dow Street, Dundee DD1 5EH, UK; 2 Institut de Biologia Evolutiva (CSIC-Universitat Pompeu Fabra), Passeig Marítim de la Barceloneta 37-49, 08003 Barcelona, Spain

**Keywords:** evolution of soma, gene duplication, transcription factor evolution, RNA-seq, cup gene expression, stalk morphogenesis, Dictyostelia, Polysphondylia, phylogenetics, cell-type specialization

## Abstract

The evolution of novel cell types has been proposed to result from duplication of gene regulatory networks, but proven examples are rare. In addition to stalk cells and spores that make up the fruiting bodies of three major groups of Dictyostelia, those in group 4 additionally evolved basal disc and cup cells that respectively anchor the stalk to the substratum and the spore mass to the stalk. We noted a putative group-4-specific duplication of a cudA-like transcription factor (TF) in a comparative analysis of group-representative genomes. Using increased taxon sampling, we here confirmed that this TF, *cdl1,* duplicated into *cdl1a* and *cdl1b* in the common ancestor to group 4. *cdl1a*, but not *cdl1b*, showed signatures of positive selection, indicative of functional innovation. Deletion of *cdl1a* in *Dictyostelium discoideum* resulted in fruiting bodies with sagging spore heads that lacked the supporting cup cells and expression of cup-specific genes. Deletion of *cdl1b* resulted in thinner fruiting body stalks, while a *cdl1b*^*−*^*cdl1a*^*−*^ double knockout showed more severe stalk defects, suggesting an ancestral role of *cdl1* in stalk formation. This was confirmed in a closely related non-group 4 species, *Polysphondylium violaceum*, where *cdl1* knockout caused defective stalk formation. These data indicate that the group-specific duplication of *cdl1* and subsequent diversification of *cdl1a* played a pivotal role in the evolution of a novel somatic cell type in group 4 Dictyostelia.

## Highlights

• The transcription factor *cdl1* duplicated into *cdl1a* and *cdl1b* in group 4 Dictyostelia
• *cdl1a* knockout results in loss of cup cells, a novel cell type in group 4
• *cdl1b* and *cdl1* knockout causes stalk defects in and outside of group 4
• Gene duplication of *cdl1* caused the evolution of a novel cell type

## Teaser

Novel cell type evolution is posited to result from duplication of gene regulatory networks. Kin et al. show that cup cells, which evolved in some Dictyostelia, require the transcription factor *cdl1A*, a novel duplicate of *cdl1*, which mediates stalk formation in all Dictyostelia. This provides evidence for cell type evolution by gene duplication.

## Introduction

Multicellularity allowed cells to specialize and perform different functions within a single organism, giving rise to two fundamental cell types, gametes and somatic cells. In several multicellular lineages, a wide range of different somatic cells evolved that act in concert to generate complex forms. How different cell types evolved is an active area of research,^1^, ^2^, ^3^ with new technologies, such as single-cell RNA sequencing (RNA-seq) facilitating the quest for homologous cell types in evolution.^4^^,^^5^ However, many of these studies are phenomenological, and there are few studies that demonstrate specific molecular mechanisms that gave rise to novel cell types (but see, e.g., Erkenbrack et al.^6^ that showed the importance of stress response for the evolution of new cell types).

A candidate mechanism for the evolution of new cell types is through gene duplication. Gene duplication is a key driver of organismal complexity,^7^, ^8^, ^9^, ^10^ and in the context of cell type evolution, it has been proposed that duplication of a gene regulatory network can lead to duplication and diversification of a cell type.^1^^,^^11^, ^12^, ^13^ However, causal evidence that links duplication of specific genes to the evolution of a novel cell type is still lacking. We here explore this issue in the dictyostelid social amoebas and report a case of gene duplication contributing to the evolution of a new cell type.

Dictyostelia are a group of protists in the supergroup Amoebozoa that can be subdivided into four major and some minor groups based on molecular data.^14^^,^^15^ The four major phylogenetic groups were simply called groups 1–4, with group 4 including the model species *Dictyostelium discoideum*. All Dictyostelia switch between free living and multicellular forms, depending on food availability. When starved, the free-living amoebas secrete chemoattractant and form multicellular aggregates that undergo morphogenesis into fruiting bodies. In most species, the fruiting bodies consist of two encapsulated cell types, the viable compact spores and the vacuolated dead stalk cells. However, in major group 4, a novel cell type, the cup cell, evolved.^16^^,^^17^

Cup cells are amoeboid cells localized in the upper and lower part of the spore mass.^18^^,^^19^ They are mainly derived from precursor cells called anterior-like cells (ALCs) and a subpopulation of prestalk cells.^19^^,^^20^ Their documented function is the elevation of spore mass: when cup cells are surgically removed, the spore mass fails to reach the top of the stalk.^21^ A recent RNA-seq study revealed that many genes became expressed in cup cells after the spore masses were already fully elevated,^22^ suggesting additional roles for cup cells.

The molecular pathways that trigger cup cell differentiation are unknown. To identify genes involved in this process, we looked for a cup-cell-specific transcription factor (TF), which is also a product of group-4-specific gene duplication. We found a possible case in the cudA-like TF family. CudA-like (cdl) proteins are TFs that are only found in Amoebozoa. Members of this family have developmental roles in *D. discoideum*, with *cudA* regulating both prespore and stalk cell differentiation^23^ and *spaA* being essential for spore differentiation.^24^

By increasing taxon sampling for molecular phylogenetics, we established that *cdl1* genes duplicated in the common ancestor to group 4, producing two copies, *cdl1a* and *cdl1b*. We found an episode of positive selection on the stem lineage of the *cdl1a* genes and revealed by gene knockout that *cdl1a* is essential for cup cell differentiation. Combined with the analyses of the knockout phenotypes of *cdl1b* as well as those of the double *cdl1b*^*−*^*cdl1a*^*−*^ knockout, we characterized the diverging functions of the duplicated genes and established the function of the ancestral *cdl1* gene in *Polysphondylium violaceum*, a sister species to group 4.

## Results

### Molecular phylogenetics reveals a *cdl1* gene duplication in group 4 Dictyostelia

To identify TFs that duplicated in group 4 Dictyostelia, we screened phylogenies in a previous evolutionary comparative study of dictyostelid TF families^25^ for clades that contain only a single TF gene from non-group 4 dictyostelid species but two or more genes in group 4 species. We found four possible duplications (or multiplications) in cudA-like, myb, Jumonji C, and homeo domain TFs. Among them, the cudA-like gene was the best candidate in the sense that the phylogenetic tree was well resolved and that one of the cudA-like duplicates was specifically expressed in cup cells, an evolutionary novelty of group 4 (Table S1).

**Figure 1:**
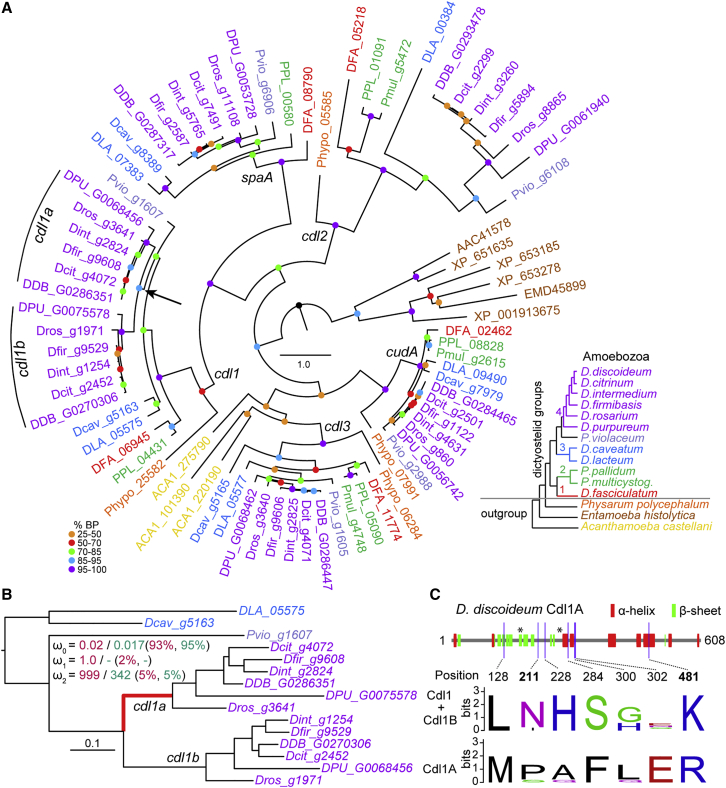
CudA-like phylogeny and cdl1 selection signatures (A) CudA-like phylogeny. Homologs of the five cudA-like genes (*cdl1*, *cdl2*, *cdl3*, *cudA*, and *spaA*) recognized in *D. discoideum* were identified from the genomes of twelve Dictyostelia and three non-dictyostelid Amoebozoa. A maximum likelihood (ML) tree was inferred from a protein sequence alignment using IQ tree with a “LG+F+R5” model. Color-coded bootstrap support values (BP) are shown on each node. Gene names are color coded according to the species of origin as in the reference Amoebozoa phylogeny shown in the inset. Sequences from dictyostelid species are denoted with the prefixes corresponding to the first three letters of species names (Pvio for *P. violaceum*, Dros for *D. rosarium*, etc.), except for “DDB_” genes from *D. discoideum* and “PPL_” genes from *P. pallidum.* The putative gene duplication event is indicated by an arrow. (B) *cdl1* DNA phylogeny. An ML tree was inferred from a codon-guided alignment of the nucleotide sequences of group 3 and 4 *cdl1* genes. The stem branch of *cdl1a* genes is shown in red. Omega (ω = dN/dS) values for negatively (ω_0_), neutrally (ω_1_), and positively (ω_2_) selected sites were estimated with codeml or aBSREL and are shown in red and green font, respectively, with the proportions of sites in each ω rate category shown in parentheses. Because aBSREL found only two rates of ω, the proportion of neutral sites (ω_1_) is not shown. (C) Sites under selection. Top: schematic of the secondary structure of Cdl1A is shown, as predicted with JPred 4, with α helices and β sheets shown as red and green rectangles, respectively. Asterisks indicate putative DNA binding sites previously identified in CudA,^26^ which are also conserved in Cdl1A. The sites found in codeml to be under positive selection (NEB > 0.95) are shown with vertical lines and text indicating their position. The two sites with BEB > 0.95 are shown in bold text. Bottom: the sequence logos of the selected sites are shown for *cdl1* and *cdl1b* genes (9 sequences) and *cdl1a* genes (6 sequences) to highlight amino acid changes with potential functional implications. See Data S2 for the amino acid and nucleotide alignments.

The previous study included only two group 4 species (*D. discoideum* and *D. purpureum*) and one species each from the other major groups.^25^ Importantly, it did not include *P. violaceum*, the closest outgroup species to group 4. We therefore reconstructed a more comprehensive phylogeny of *cudA*-like genes, using recently sequenced genomes (Figure 1A). As noted before,^25^ *cudA*-like genes were only found in Amoebozoa and are subdivided into five ortholog groups. Two genes, *cudA* and *spaA*, have essential roles in stalk and spore differentiation in *D. discoideum*, respectively.^23^^,^^24^ We designated the uncharacterized genes as *cdl* for cudA-like. All five genes are found in most of the dictyostelid genomes, except for *cdl1* and *spaA* in *P. multicystogenum* and *cdl2* in *D. caveatum*. Their absence may be due to lineage-specific loss or poor quality of these draft genomes. The non-dictyostelid *Physarum polycephalum* possesses all *cudA*-like genes except *spaA*, while the phylogenetic affinities of the *Entamoeba* and *Acanthamoeba cudA*-like genes to the five groups are less clear.

The new phylogeny corroborates duplication of a gene named *cdl1* into the paralogs, *cdl1a* and *cdl1b*, which are present in all six examined group 4 species. Only the single *cdl1* gene exists in non-group 4 species, including the closest outgroup *P. violaceum*. This phylogenetic distribution suggests that the duplication of *cdl1* occurred in the stem lineage of group 4. Interestingly, *cdl1*/*cdl1a* was found in close genomic proximity to *cdl3* in all group 4 and group 3 species (Table 1), but not in group 1 or 2 species. In groups 3 and 4, *cdl1*/*cdl1a* and *cdl3* show the same orientation, with *cdl1*/*cdl1a* usually 3 to 4 kb upstream of *cdl3* but sometimes less than 0.3 kb. This indicates that the genomic linkage of *cdl1* and *cdl3* evolved after the split of groups 1 and 2 from groups 3 and 4. The linkage of *cdl1* or *cdl1a* with *cdl3* further suggests that *cdl1b* evolved by transposition of the *cdl1* gene to a separate genomic location after the split of group 3 and group 4 (in *D. discoideum* from chromosome 4 to chromosome 1).

**Table 1:** Genome locations of *cdl1*/*cdl1a* genes and *cdl3* genes

Species	Contig	Upstream	Distance (bps)
**Groups 3 and 4**			

*D. discoideum*	Chromosome 4	*cdl1a*	4,379
*D. purpureum*	DPU0000507	*cdl1a*	4,271
*D. rosarium*	Dr_05690	*cdl1a*	150
*D. citrinum*	JH790292	*cdl1a*	264
*D. firmibasis*	JH723829	*cdl1a*	4,669
*D. intermedium*	JH722772	*cdl1a*	4,367
*P. violaceum*	AJWJ01000039	*cdl1*	3,803
*D. caveatum*	Dc_12289	*cdl1*	3,499
*D. lacteum*	GAOABQK02HUB3S	*cdl1*	3,150

**Groups 1 and 2**			

*P. pallidum*	Different	N/A	N/A
*P. multicystogenum*	N/A	N/A	N/A
*D. fasciculatum*	Different	N/A	N/A

In groups 3 and 4 dictyostelids, *cdl1* or *cdl1a* and *cdl3* are always on the same contig and in the same order (*cdl1*/*cdl1a* being upstream) on the genome. *cdl1b* is in *D. discoideum* located on chromosome 1.

### A signature for positive selection in the *cdl1a* stem lineage

Duplicated genes can experience various patterns of natural selection and undergo functional divergence.^27^ In particular, adaptive evolution is expected to occur when a gene acquires a new function during evolution. To test whether either of the *cdl1* duplicates experienced adaptive evolution, we fitted the branch-site model where the nonsynonymous-synonymous substitution rates (ω = dN/dS) can vary across both lineages and sites during evolution. Two different models, M2a (branch-site-positive selection model) in codeml^28^^,^^29^ and adaptive branch-site random effects likelihood (aBSREL) in Hyphy^30^^,^^31^ gave broadly the same results (Figure 1B; Table 2). In both cases, the alternative model with positively selected sites in the stem lineage of *cdl1a* had significantly higher likelihoods compared to the null model without positively selected sites. In contrast, the alternative model of the stem lineage of *cdl1b* being positively selected was not supported.

**Table 2:** Parameters estimated in the selection tests in the *cdl1a* and *cdl1b* stem lineage

Model	−2 Δ	p value	Parameters	Proportion
**cdl1a**				

Codeml (M2a)	16.1	6.0 × 10^−5^	ω0 = 0.2, ω1 = 1, ω2 = 999	p0 = 0.93, p1 = 0.02, p2a = 0.049, p2b = 0.001
aBSREL	15.2	5.0 × 10^−3^	ω0 = 0.02, ω2 = 342	p0 = 0.948, p2 = 0.052

**cdl1b**				

Codeml (M2a)	0.14	0.7	ω0 = 0.02, ω1 = 1, ω2 = 3.4	p0 = 0.74, p1 = 0.02, p2a = 0.23, p2b = 0.01
aBSREL	0.66	1.0	ω0 = 0.124, ω2 = 101	p0 = 0.976, p2 = 0.024

From left to right: (1) the models used, (2) the differences in log-likelihoods between the null and the alternative models (Δ) multiplied by −2, (3) p values (for aBSREL, adjusted p values corrected for multiple testing are shown), (4) the estimated dN/dS (ω) parameters (ω_0_ for negatively selected, ω_1_ for neutral, and ω_2_ for positively selected sites), and (5) the proportion of sites under each category.

The codeml analysis identified seven amino acid residues in Cdl1A as sites possibly under positive selection, with naive empirical Bayes (NEB) posterior probability above 0.95. Two of them were also supported with Bayes empirical Bayes (BEB) posterior probability being above 0.95. Although the structure of the Cdl1A protein has not been resolved, many of the positively selected sites were located on or close to predicted α helices or β sheets (Figure 1C). Amino acid residues that are essential for DNA binding in CudA^26^ are also conserved in Cdl1A (asterisks in Figure 1C). These sites are distinct from the positively selected sites but are within a 174 AA (amino acid) region that contains 6 out of 7 putative sites.

### *Cdl1a* is essential for cup cell differentiation

**Figure 2:**
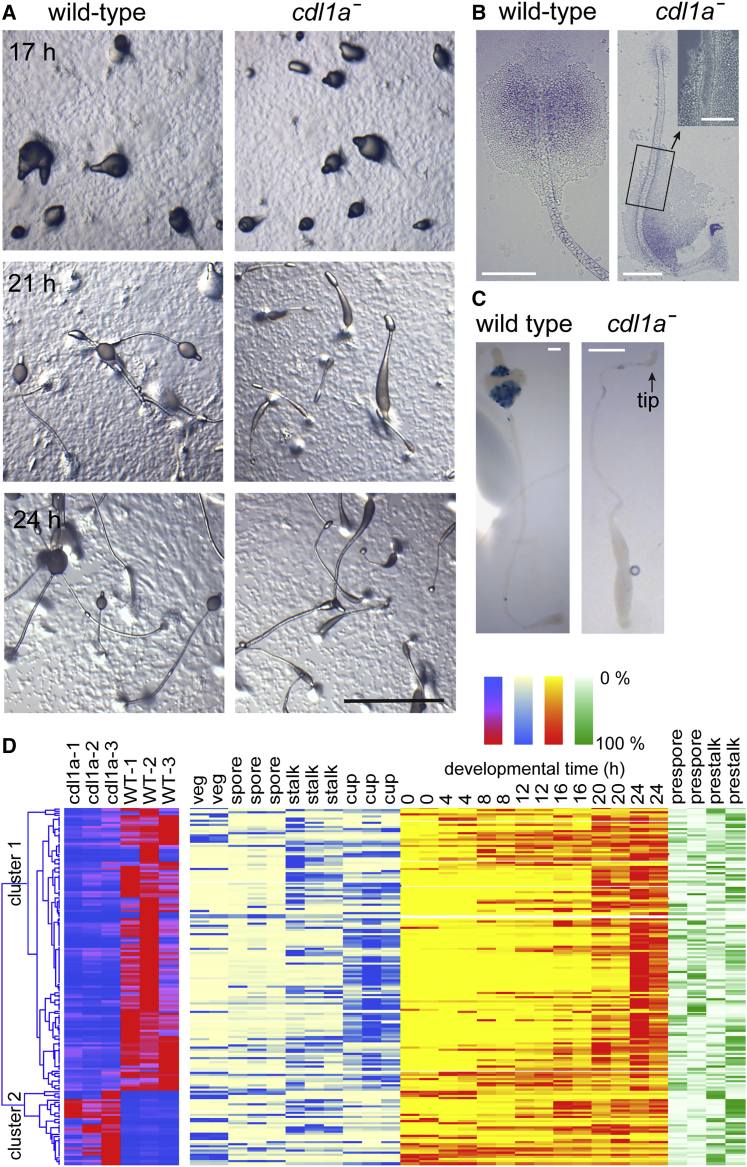
Phenotype of *cdl1a* knockout mutant (A) Development. *Ddis cdl1a* was knocked out by homologous recombination (see Figure S1A for a schematic and PCR diagnosis of the knockout). Wild-type and *cdl1a*^*−*^ cells were incubated on non-nutrient agar for 24 h and imaged from early culmination (17 h) onward with a dissecting microscope. See Video S1 for culmination of *cdl1a*^*−*^ fruiting bodies. Scale bar, 1 mm. (B) Stalk and spores. Mature wild-type and *cdl1a*^*−*^ fruiting bodies were squashed under a coverslip and imaged with a compound microscope. Scale bars: main image, 100 μm; inset, 50 μm. (C) Cup gene expression. Wild type and *cdl1a*^*−*^, transformed with *p[beiA]:lacZ*, were developed into fruiting bodies, stained with X-gal, and imaged with a compound microscope. Scale bars, 100 μm. See also Figure S2 for expression of the stalk, cup, and basal disc markers *ecmA* and *ecmB*. (D) RNA-seq. mRNAs of maturing wild-type (WT) and *cdl1a*^*−*^ fruiting bodies were isolated from three separate experiments and sequenced on the Illumina platform. 170 genes were differentially expressed between WT and *cdl1a*^*−*^ at FDR < 0.05. The transcript levels of the differentially expressed genes were standardized to percentage of maximum, ordered by hierarchical clustering, and are shown in the blue and red heatmap on the left. The same genes were further annotated with heatmaps of published developmental (yellow-red) and cell-type-specific transcripts (pale yellow-blue and white-green)^22^^,^^33^ in the panels to the right. These transcripts were also standardized to percentage of maximum reads or for prespore and prestalk comparisons as percentage of summed reads. See Data S1 for the full RNA-seq analysis. See also Table S1 and Figures S1 and S2.

To investigate a possible role for Cdl1A, we knocked out the *cdl1a* gene in *D. discoideum* (Figure S1A). The *cdl1a*^*−*^ cells formed normal aggregates and slugs but showed aberrant fruiting body formation (Figure 2). At early culmination, the prespore mass did not properly follow the elevation of the tip (Figure 2A; Video S1), resulting in fruiting bodies with the spore heads stuck at the base or middle of the stalk. Only a small mass of what are likely prestalk cells were carried aloft. Spore and stalk cells otherwise differentiated normally (Figure 2B). The impaired elevation of the spore mass was also observed when cup cells were surgically removed.^21^ We therefore examined the presence of cup cells in *cdl1a*^*−*^ by observing expression of *lacZ* fused to the promoter of the cup-specific gene *beiA*.^22^^,^^32^ *[beiA]:lacZ*-expressing cells were almost completely absent from *cdl1a*^*−*^ fruiting bodies, suggesting a lack of cup cells (Figure 2C).

Video S1. A time-lapse movie of the culmination of *cdl1a*^*−*^, related to Figure 2

To obtain a comprehensive overview of genes not expressed in *cdl1a*^*−*^, we performed high-throughput sequencing of RNAs isolated from late wild-type and *cdl1a*^*−*^ fruiting bodies. Compared to wild type, 135 genes were significantly (false discovery rate [FDR] < 0.05) underexpressed in *cdl1a*^*−*^ and 35 genes were overexpressed (see Data S1 for data analysis and gene annotation). Differentially expressed transcripts, standardized to maximum read counts, for the three replicate experiments are shown in Figure 2D as red-blue heatmaps and are combined with heatmaps of published RNA-seq data for the same genes of purified cell types and developmental time points of wild-type *D. discoideum* cells.^22^^,^^33^ This comparison shows that the top cluster of genes, downregulated in *cdl1a*^*−*^, are in wild-type *D. discoideum* mostly upregulated in late development and enriched in cup cells, with about half of the genes also or alternatively upregulated in stalk cells. This substantiates the evidence that Cdl1A is a transcription factor, required for cup gene expression. There is no obvious preference for prestalk or prespore expression of the genes downregulated in *cdl1a*^*−*^*.* The genes upregulated in *cdl1a*^*−*^ are generally not expressed in cup cells and tend to be expressed throughout development. Contrary to notions that cup cells are derived from prestalk cells, the upregulated genes in the cupless *cdl1a*^*−*^ mutant were mostly prestalk specific.

We also examined the expression pattern of the prestalk and stalk marker genes *ecmA* and *ecmB*,^34^ which, apart from being expressed in the stalk and basal disc, are also expressed in cup cells. The *ecmA* or *ecmB* expressing cup precursors are in wild type derived from so-called anterior-like cells (ALCs) that are scattered among the posterior prespore cells in the slug and move toward the upper and lower cup positions during culmination.^19^ Up to the early culminant stage, the spatial expression patterns of *ecmA* and *ecmB* were not markedly different between wild type and *cdl1a*^*−*^ (Figures S2A and S2B). Thereafter, both genes showed normal expression in the *cdl1a*^*−*^ prestalk and stalk cells. However, instead of accumulating in the cup region, cells expressing *ecmA* or *ecmB* remained intermixed with spores. This suggests that the *ecmA* and *ecmB* expressing cup precursors were initially formed but never committed to cup differentiation. After prolonged incubation (>2 days), secondary sorogens often emerged from the sagging spore masses of *cdl1a*^*−*^ (Figure S2C), which are possibly formed from the uncommitted cup precursors, because all the remaining cells are at this stage encapsulated as spores or stalk cells. Neither *ecmA* nor *ecmB* transcripts were significantly differentially expressed between wild-type and *cdl1a*^*−*^ fruiting bodies, and transcript numbers were actually somewhat higher in *cdl1a*^*−*^ (Figure S2D), indicating that these genes are not positively regulated by Cdl1A.

The strong association between the presence of Cdl1A, the expression of cup-specific genes, and the presence of cup cells indicates that Cdl1A is a transcription factor that activates expression of genes essential for cup differentiation.

### *Cdl1a* is expressed in stalk and cup precursors

**Figure 3:**
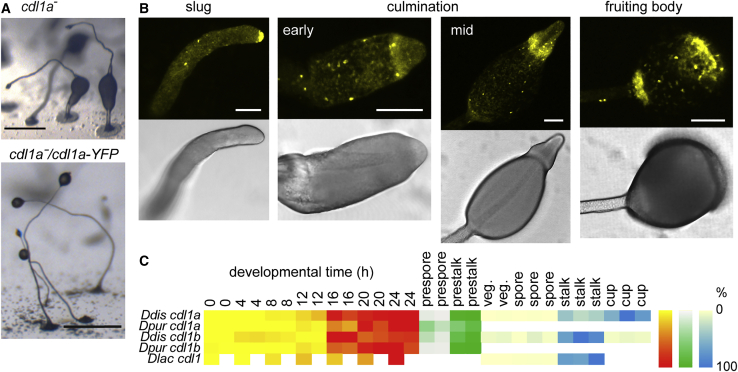
*cdl1a*^*−*^ complementation and *cdl1a* expression (A) *cdl1a*^−^ complementation. The *cdl1a* gene and its promoter were fused at the C terminus to YFP and transformed into *cdl1a*^*−*^ cells. The transformed cells and their parent were developed into fruiting bodies and imaged with a dissecting microscope. Scale bars, 0.5 mm. (B) *Cdl1A-YFP* expression. The localization of the *Cdl1A-YFP* protein was visualized by confocal microscopy at the indicated stages, with transilluminated structures shown in the bottom panels. Scale bars, 100 μm. (C) *cdl1* transcripts. Published RNA-seq data of *D. discoideum*, *D. purpureum*, and *D. lacteum* developmental time courses and purified cell types^25^^,^^33^^,^^35^ were standardized as percentage of summed (prestalk and prespore) or maximum (all others) read counts of a series and displayed as heatmaps.

To visualize the expression pattern of Cdl1A, the *cdl1A* gene inclusive of the 3 kb 5′ intergenic sequence was fused to YFP (yellow fluorescent protein) and transformed into *cdl1a*^*−*^ cells. The *p[cdl1a]:cdl1a-YFP* construct restored the normal uplift of spores in *cdl1a*^*−*^ (Figure 3A), confirming that the loss of cup cells in *cdl1a*^*−*^ was due to the gene knockout. Cdl1A-YFP was initially expressed strongly at the slug tip and in cells scattered throughout the slug. During culmination, expression became more pronounced at the prespore and prestalk boundary and in the lower cup region until it was almost completely confined to the upper and lower cup in mature fruiting bodies (Figure 3B).

The temporal expression pattern and cell type specificity of *cdl1a* and *cdl1b* and its ancestor *cdl1* were also inferred from published RNA-seq experiments of *D. discoideum*, *D. purpureum*, and *D. lacteum*,^25^^,^^33^^,^^35^ which show that *cdl1a* and *cdl1b* expression is upregulated at 16 h when migrating slugs have formed (Figure 3C). Expression of both genes is highest in the prestalk cells, and while *cdl1b* expression then remains confined to the stalk cells, *cdl1a* becomes more strongly expressed in the cup cells. The ancestral *cdl1* gene is also upregulated in late development and is specific to stalk cells in *D. lacteum*. Evidently, the novel role of Cdl1A as cup cell inducer involved elaboration of its ancestral expression pattern in stalk cells.

### Deletion of both *cdl1a* and *cdl1b* causes defects in stalk morphogenesis

**Figure 4:**
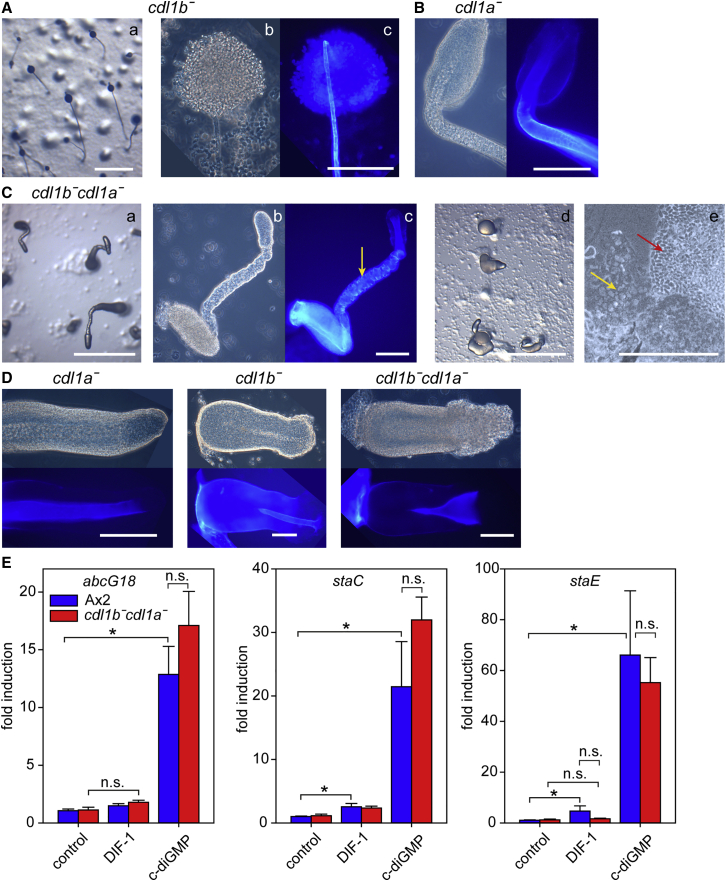
Fruiting body morphology of *cdl1b*^*−*^ and *cdl1b*^*−*^*cdl1a*^*−*^ (A) *cdl1b*^−^ mutant. The *cdl1b* gene was knocked out by homologous recombination (Figure S1B). *cdl1b*^*−*^ cells were developed into fruiting bodies and imaged *in situ* with a dissecting microscope (a) or transferred to a slide glass, stained with the cellulose dye Calcofluor, and imaged with a compound microscope (b: phase contrast; c: fluorescence). Scale bars: Aa, 1 mm; other panels, 100 μm. (B) *cdl1a*^−^ mutant. The *cdl1a*^*−*^ mutant was developed, stained with Calcofluor, and imaged as described above with a compound microscope, showing normal stalk formation at the tip (a residual prestalk cell mass can be seen, but the spore mass is lower down). Scale bar, 100 μm. (C) *cdl1b*^*−*^*cdl1a*^*−*^ mutant. *Cdl1a* was deleted in the *cdl1b*^*−*^ mutant (Figure S1A), and the resulting *cdl1b*^*−*^*cdl1a*^*−*^ cells were developed into fruiting bodies, stained with Calcofluor, and imaged as described above. Severe (a–c) to very severe (d and e) disruptions of terminal fruiting body morphology were observed, but spores (red arrow) and disorganized stalk cells (yellow arrows) were still present. Scale bars: Ca, 1 mm; other panels, 100 μm. (D) Early stalk formation. The newly formed stalk of the *cdl1a*^*−*^, *cdl1b*−, and *cdl1a*^*−*^*cdl1b*^*−*^ mutants was visualized by phase contrast and Calcofluor staining. Scale bars, 100 μm. (E) Stalk gene induction. Ax2 and *cdl1b*^*−*^*cdl1a*^*−*^ mutant cells were developed to first fingers, dissociated, and incubated without additives (control) or with 100 nM DIF-1 or 3 μM c-di-GMP. After 6 h, RNAs were isolated and used as templates for qRT-PCR, using primers specific to the stalk genes *abcG18*, *staC* (DDB_G0271196),^32^ and *staE* (DDB_G0287091)^22^ and two constitutively expressed genes (DDB_G0282429 and/or DDB_G0280765). Normalized data are shown as fold induction over the wild-type control and represent means and standard error (SE) of two or three experiments performed in triplicate. Results from informative combinations of variables were tested for significant differences with a t test or rank sum test, with ^∗^ marking significant (p < 0.05) and n.s. non-significant differences. See also Figure S1.

To examine functional redundancy or a divergent role for *cdl1b*, we deleted first *cdl1b* and then *cdl1b* and *cdl1a* together. The *cdl1b*^*−*^ mutants developed normally and produced apparently normal fruiting bodies, but their stalks were often thinner, compared to wild type or *cdl1a*^*−*^, as is evident by staining with Calcofluor, which reacts with cellulose in the stalk and spore walls and the tube that surrounds the stalk (Figures 4A and 4B). The *cdl1b*^*−*^*cdl1a*^*−*^ strain developed normally until culmination, but then proper stalk formation was impaired, with phenotypes ranging from severe to very severe. In a severe case, stalk-like structures projected upward but with a sagging spore mass like *cdl1a*^*−*^ (Figures 4Ca–4Cc). The stalky part of this projection contained vacuolized cells staining with Calcofluor, but its shape was irregular. While a cellulosic stalk tube was formed, it appeared to no longer constrain most of the stalk cells (Figure 4Cc). In a very severe case, the structures showed only minimal upward projection and consisted of disorganized stalk cells and a spore mass (Figures 4Cd and 4Ce).

The aberrant stalk formation was already evident at the onset of culmination. In *cdl1b*^*−*^, the stalk tube is rather thin from the outset, while in *cdl1a*^*−*^*cdl1b*^*−*^, the opening of the stalk tube is very wide, giving it a funnel-like appearance (Figure 4D). Overall, Cdl1B and Cdl1A seem to be required together for proper stalk morphogenesis, rather than stalk cell differentiation. To further test this notion, we quantitatively compared induction of the stalk genes *abcG18*, *staC*, and *staE* by the stalk-inducing signals DIF-1 (differentiation inducing factor 1) and c-di-GMP between wild type and *cdl1a*^*−*^*cdl1b*^*−*^ by qRT-PCR (Figure 4E). Effects of DIF-1 were relatively small in both wild type and *cdl1a*^*−*^*cdl1b*^*−*^, but c-di-GMP induced stalk gene expression over 15-fold, with no significant difference being evident between the two strains. While it is still possible that Cdl1A and Cdl1B regulate subsets of stalk genes, a role in overt stalk cell differentiation is less likely.

### *Polysphondylium violaceum* Cdl1 is required for stalk morphogenesis

**Figure 5:**
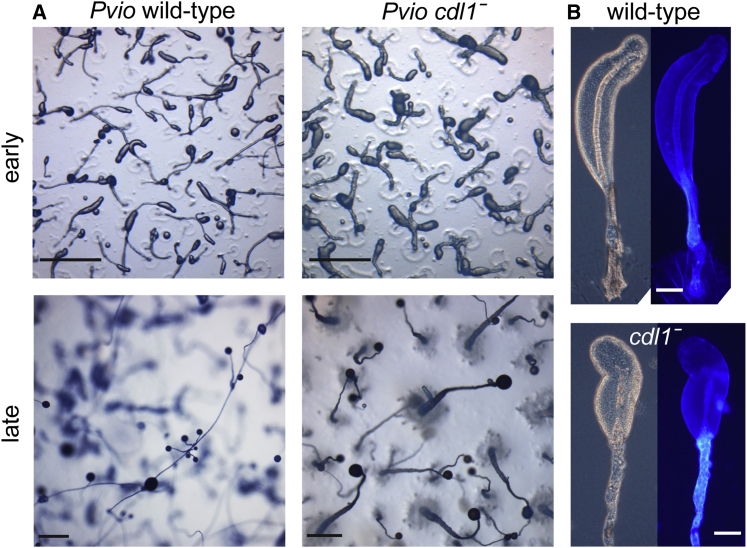
Phenotype of *P. violaceum cdl1*^*−*^ (A) Development. The *P. violaceum cdl1* gene was deleted by homologous recombination (Figure S1C), and *cdl1*^*−*^ and wild-type *P. violaceum* were developed into fruiting bodies on non-nutrient agar. Early culminants (top) and mature fruiting bodies (bottom) were imaged *in situ* with a dissecting microscope. Scale bars, 0.5 mm. (B) Stalk formation. Early culminants were also carried over to a slide glass, stained with Calcofluor, and imaged with a compound microscope (left: phase contrast; right: fluorescence). Scale bars, 50 μm. See also Figure S1.

The gene duplication that generated *cdl1a* and *cdl1b* occurred early in the group 4 lineage (Figure 1). To examine the ancestral function of their proto-ortholog *cdl1*, we knocked out *cdl1* in *P. violaceum*, the closest outgroup species to group 4. The *P. violaceum cdl1*^*−*^ cells developed normally up to early culmination, when stalk formation initiates. The *cdl1*^*−*^ culminants produced thicker, more rugged stalks than wild type, and the cell masses that were carried aloft tended to have a more bulbous or curvy appearance. The mature *cdl1*^*−*^ fruiting bodies were significantly shorter than those of wild type and almost entirely failed to produce the whorls of side branches that typify the Polysphondylia (Figure 5A). When stained with Calcofluor, the *cdl1*^*−*^ mutant showed an irregular and sometimes fragmented arrangement of stalk cells in the sorogen (Figure 5B).

## Discussion

### A group-4-specific gene duplication associated with cup cell emergence

Duplication of genes in gene regulatory networks was proposed as a possible mechanism for the evolution of novel cell types in multicellular organisms. Group 4 Dictyostelia evolved large robust fruiting bodies with new cell types and other innovations that are not present in the other groups.^16^^,^^17^ To identify a putative regulatory network that duplicated in group 4, we screened phylogenies of the ∼440 dictyostelid TFs across taxon group representative species^25^ for TFs that were duplicated in group 4. The clearest example specific to group 4 was found in the cudA-like (*cdl*) family. Sampling of *cdl* homologs from four additional group 4 and three more non-group 4 species corroborated that the *cdl1* duplication occurred after the common ancestor to group 4 split from its small sister group, which contains *P. violaceum* (Figure 1A). The genomic proximity of *cdl1* genes to *cdl3* allowed us to infer that one copy, *cdl1b*, was produced by a copy-and-paste type duplication of *cdl1* to another chromosome, while the other, *cdl1a*, can be considered as the “original” copy (Table 1). *Cdl1a* showed evidence of having experienced positive selection on seven different sites, which could have contributed to the evolution of a derived function of this gene (Figures 1B and 1C).

Deletion of *D. discoideum cdl1a* prevented the differentiation of cup cells in the fruiting body as well as the expression of many cup-specific genes (Figure 2), indicating that Cdl1A plays a decisive role in the induction of cup cell differentiation. Consistent with effects of surgical removal of cup cells,^21^ the spore mass of *cdl1a*^*−*^ failed to elevate to the top of the stalk.

### Combinatorial control of stalk morphogenesis by Cdl1A and Cdl1B

Deletion of the *cdl1a* duplicate *cdl1b* caused more subtle effects; fruiting bodies were formed normally but appeared to have thinner stalks. When both *cdl1a* and *cdl1b* were knocked out, the stalk defects were more severe. The cellulose stalk tube was formed but showed abnormalities, and the arrangement of the stalk cells inside the tube was disorganized. Stalk genes were still induced by the stalk-inducing factor c-di-GMP, so the *cdl1a* and *cdl1b* lesions appeared to affect stalk morphogenesis more than stalk cell differentiation (Figure 4). While *cdl1a*^*−*^ cells do not show obvious stalk defects, *cdl1a* is expressed at the slug tip in addition to the cup cells (Figure 3B). A synergistic role with *cdl1b* in stalk formation is therefore not unexpected and suggests that the ancestral *cdl1* gene was involved in stalk morphogenesis. Consistent with this hypothesis, knockout of *cdl1* in an outgroup species, *P. violaceum*, also caused defects in stalk morphogenesis. The newly formed stalk tube in the *P. violaceum cdl1*^*−*^ mutant appeared to be fragmented. Stalk cells still differentiated but were more disordered, resulting in short, rugged stalks (Figure 5). This indicates that the ancestral function of *cdl1* in the Dictyostelia was to form proper stalks. During the evolution of group 4, this gene duplicated and one of the duplicated genes, *cdl1a*, became additionally involved in inducing the novel cup cells of this group.

While a role for gene duplication in the evolution of novel cell types has been repeatedly pointed out,^1^^,^^11^, ^12^, ^13^ no empirical examples are known, apart from a few studies in fungi that relate gene duplication to evolution of new gene regulatory networks, albeit not cell type.^36^, ^37^, ^38^ Our study is one of the first examples where gene duplication of a transcription factor and the evolution of a new cell type are causally linked, although many questions remain. How did the duplicated gene evolve to acquire its novel function? What are the upstream TFs and signaling molecules that regulate the *cdl1* genes? How are the regulatory programs different between the pre-duplication gene *cdl1* and the post-duplication gene, *cdl1a*? Answering these questions in future will provide better mechanistic insights in the evolution of novel cell types.

## STAR★Methods

### Key resources table

**Table:** 

REAGENT or RESOURCE	SOURCE	IDENTIFIER
**Chemicals, peptides, and recombinant proteins**		

Blastcidin	InvivoGen	Cat#ant-bl-1
G418	ThermoFisher	Cat#11811023
HL5 axenic medium	Formedium	Cat#HLG0101
1/5th SM agar	Formedium	Cat#SMA50101
Glutaraldehyde	Sigma	Cat#111-30-8
Bacto-Agar	BD	Cat#214010
X-gal	Sigma	Cat#7240-90-6
DIF-1	Enzo Life Sciences	Cat#BML-GR324-0100
c-di-GMP	Biolog	Cat#C057-01
Calcofluor	Sigma	Cat#910090-20ML

**Critical commercial assays**		

NEBNext Ultra II Directional RNA Kit	New England Biolab	Cat#E7760S
Agilent TapeStation DNA D1000 HS Kit	Agilent	Cat# 5067-5585
RNeasy Mini Kit	QIAGEN	Cat#74104
Turbo DNA-free kit	Invitrogen	Cat#AM1907
SensiFAST™ cDNA synthesis kit	Bioline	Cat#BIO-65053
PerfeCTa SYBR Green SuperMix	QuantaBio	Cat#95053-500

**Deposited data**		

Raw RNA-Seq reads *cdl1aˉ* and wildtype *D. discoideum AX2*	This paper	ENA: PRJEB46667

**Experimental models: organisms/strains**		

*Dictyostelium discoideum* AX2	G. Gerisch, MPI Biochemie, Munich	dictybase: DBS0237914
*Polysphondylium violaceum* Qsvi11	Kalla et al.^39^	dictyBase: DBS0350965
*Dictyostelium discoideum cdl1aˉ*	This paper	dictybase: DBS0351684
*Dictyostelium discoideum cdl1bˉ*	This paper	dictybase: DBS0351685
*Dictyostelium discoideum cdl1aˉ/cdl1bˉ*	This paper	dictybase: DBS0351690
*Polysphondylium violaceum cdl1ˉ*	This paper	dictybase: DBS0351688

**Oligonucleotides**		

Primers for constructing knockout and expression vectors in *D. discoideum* and *P. violaceum*, see Table S2	This paper	N/A

**Recombinant DNA**		

PLPBLP	Faix et al.^40^	dictyBase: DBP0000009
pDEX-NLS-cre	Faix et al.^40^	dictyBase: DBP0000008
pLoxP-NeoIII	Kawabe et al.^41^	dictyBase: 1085
pB17S-EYFP	C.J. Weijer, Dundee University	dictyBase: 1084
pCdl1a-KO	This paper	dictyBase: DBP0001075
pCdl1b-KO	This paper	dictyBase: DBP0001076
pPvio-cdl1-KO	This paper	dictyBase: DBP0001080
p[cdl1a]:cdl1a-YFP	This paper	dictyBase: 1082
pEcmA-ile-gal	Detterbeck et al.^42^	dictyBase: 1083
pEcmB-gal	Ceccarelli et al.^43^	dictyBase: DBP0000051
p[beiA]:lacZ (dictyBase: pDd17-gal-DDB_G0276063)	Chen et al.^32^	dictyBase: DBP0001037

**Software and algorithms**		

ImageJ	Schneider et al.^44^	https://imagej.nih.gov/ij/
FastQC	Babraham Bioinformatics resources	https://www.bioinformatics.babraham.ac.uk/projects/fastqc/
RSEM	Li and Dewey^45^	https://github.com/deweylab/RSEM
DESeq2	Love et al.^46^	https://bioconductor.org/packages/release/bioc/html/DESeq2.html
MAFFT	Katoh and Standley^47^	https://mafft.cbrc.jp/alignment/software/
IQ-TREE	Nguyen et al.^48^	http://www.iqtree.org/
codeml (PAML)	Yang^28^	http://abacus.gene.ucl.ac.uk/software/paml.html
aBSREL (Hyphy)	Smith et al.^31^	http://www.hyphy.org/
JPred4	Drozdetskiy et al.^49^	https://www.compbio.dundee.ac.uk/jpred/
WebLogo	Crooks et al.^50^	https://weblogo.berkeley.edu/logo.cgi

**Other**		

Dialysis membrane	Medicell Memberanes LtD	DTV12000.13.15

### Resource availability

#### Lead contact

Further information and requests for resources and reagents should be directed to and will be fulfilled by the lead contact, Pauline Schaap (p.schaap@dundee.ac.uk).

#### Materials availability

All plasmid constructs and knock-out cell lines have been deposited in the Dicty Stock Centre (http://dictybase.org/StockCenter/StockCenter.html). The assigned IDs are listed in the Key resources table.

### Experimental model and subject details

*Dictyostelium discoideum, strain* AX2, was grown at 22°C in HL5 axenic medium (Formedium, CAT# HLG0101) and *Polysphondylium violaceum, strain* Qsvi11 strain was grown at 22°C in association with *Klebsiella aerogenes* on 1/5th SM agar (Formedium, Cat#SMA50101). Mating type of cells is either not known or not relevant in this study of asexual development.

### Method details

#### Phylogenetics and detection of positive selection

The previously identified CudA-like genes^25^ were supplemented with genes identified by tblastn in draft genome assemblies of *Dictyostelium citrinum* (GCA_000286055.1), *Dictyostelium intermedium* (GCA_000277465.1), *Dictyostelium firmibasis* (GCA_000277485.1), *Dictyostelium rosarium* (GCA_013375675.1), *Dictyostelium caveatum* (GCA_003667305.1), *Polysphondylium violaceum* (GCA_000277445.1), *Polysphondylium multicystogenum* (GCA_003667245.1), *Acanthamoeba castellanii* (GCA_000313135.1) and *Entamoeba histolytica* (GCA_000208925.2), which were retrieved from GenBank (https://www.ncbi.nlm.nih.gov/genbank/). Sequences of *Physarum polycephalum* were downloaded from the *Physarum polycephalum* Genome Resource website (https://www.regulationsbiologie.ovgu.de/Downloads/Physarum+polycephalum+Genome+Resource.html). The amino acid sequences of the collected genes were aligned with the “E-INS-i” option of MAFFT (ver. 7.429)^47^ and poorly aligned sections were removed manually. The final alignment was then used for maximum likelihood (ML) phylogenetic reconstruction with IQ-tree^48^ under the model “LG+F+R5,” which was selected with ModelFinder.^51^ One hundred bootstrap replicates were generated to provide support values to the ML tree.

Signature of positive selection was explored using both codeml in PAML^28^ and aBSREL in Hyphy.^30^^,^^31^ Codon-based alignments for group 3 and 4 Dictyostelid *cdl1* genes were computed and unreliable sites were removed using Guidance 2 with the Prank alignment algorithm using default settings.^52^, ^53^, ^54^ All the sites that contain gaps were also removed. The final codon-based alignment of 15 DNA sequences with 753 nucleotides (251 codons) was used to reconstruct a ML tree of *cdl1* genes with IQ-tree under the model “GTR+F+R3” selected with ModelFinder. This tree was used as an input tree for codeml and aBSREL. In codeml, a branch-site model (model = 2, NSsites = 2 in the control file) with the codon frequency F3X4 (individual nucleotide frequencies for three codon positions) was used for computing the likelihood of the data under the M2a model and the null model with ω_2_ = 1 fixed.^29^ The log-likelihood ratios (Δ) of the alternative and null models were calculated and the p values were estimated by the right-tail probability of the test statistic −2 Δ under the χ^2^ (d.f. = 1) distribution. The aBSREL was run with default settings by providing the codon-based alignment and an input tree to the program. The secondary structure of the Cdl1A protein was predicted with JPred4.^49^ Sequence logos of the cdl protein alignments were generated with WebLogo.^50^ The full and trimmed alignments produced in the study are available in Data S2.

#### Induction and imaging of development

To induce development, *D. discoideum* or *P. violaceum* cells were harvested from growth medium or *Klebsiella* lawns, respectively, washed with 10 mM Na/K-phosphate buffer, pH 6.5 (PB) and incubated on non-nutrient (NN) agar (1.5% agar in 8.8 mM KH_2_PO_4_ and 2.7 mM Na_2_HPO_4_) at 1∼3 × 10^6^ cells/cm^2^ at 22°C, or on dialysis membrane supported by NN agar.

Developing structures on NN agar plates were imaged using a Leica MZ16 dissection microscope. Video S1 was obtained by placing a small section of agar with an early culminant of *cdl1aˉ* in mineral oil to prevent drying out, and by acquiring images with the dissection microscope at 1 min intervals for 12 h.

#### DNA constructs and transformation

To knock out *cdl1a* (*DDB_G0286351*) and *cdl1b* (*DDB_G0270306*) in *D. discoideum* and *cdl1* (Pvio_g1607, GenBank: KAF2077098) in *P. violaceum*, two fragments of each gene were amplified from *D. discoideum* or *P. violaceum* genomic DNAs and cloned into PLPBLP^40^ for *D. discoideum cdl1a* and *cdl1b*, and pLoxP-NeoIII^41^ for *P. violaceum cdl1*, using restriction sites that were introduced in the oligonucleotide primers. The primer sequences are listed in Table S2 by the gene name followed by A, B, C or D, with the A/B primer combination used for amplifying the 5′ KO1 fragment and the C/D combination the 3′ KO2 fragment.

The knock-out plasmids were linearized and introduced into *D. discoideum* Ax2 or *P. violaceum* Qsvi11 by electroporation.^55^ Transformed *D. discoideum* clones were selected by including 10 μg/ml blasticidin in HL5 growth medium. Transformed *P. violaceum* clones were selected by growth on lawns of G418-resistant *Escherichia coli* in the presence of 50 μg/ml G418.^55^ Genomic DNAs were isolated and tested for gene knock-out by two sets of PCR reactions. *D. discoideum cdl1a* and *cdl1b* clones were screened by primer pairs cdl1awf/cdl1awr and cdl1bwf/cdl1bwr for absence and by BsrF1/cdl1aKOr and BsrF1/cdl1bKOr for presence of knock-out, respectively (Figures S1A and S1B). Likewise, *P. violaceum* clones were screened by primer pairs Pvcdl1wf/Pvcdl1wr and *Pvcdl1*KOf/NeoR for absence and presence of knock-out, respectively. To construct a *cdl1bˉcdl1aˉ* double knockout, the *Bsr* cassette in *cdl1bˉ* was removed by transformation with pDEX-NLS-cre,^40^ and a blasticidin sensitive clone was transformed with the *cdl1a* knockout construct and selected for *cdl1a* deletion as above (Figure S1A).

To generate a *D. discoideum cdl1a* expression construct, the 3.08 kb *cdl1a* 5′ intergenic region was amplified from Ax2 genomic DNA with primer pair cdl1aPRf/cdl1aPRr and the *cdl1a* coding region with primer pair cdl1aCDSf/cdl1aCDSr (Table S2). The fragments were sequentially inserted into pB17S-EYFP,^56^ using the restriction sites that were introduced into the primers. This yielded plasmid p[cdl1a]:cdl1a-YFP, which was introduced into *D. discoideum* by electroporation. Transformed clones were selected by growth in the presence of 10 μg/ml G418.

#### Detection of β-galactosidase or YFP in developing structures

Dialysis membranes with developing structures of cells harboring promoter-*lacZ* gene fusions were transferred to filter paper soaked in 0.5% glutaraldehyde, incubated in a sealed chamber for 3 min, and then submersed in 0.5% glutaraldehyde for 3 min. After washing with Z-buffer (60 mM Na_2_HPO_4_, 40 mM NaH_2_PO_4_, 10 mM KCl, 1 mM MgSO_4_, pH 7.0), cells were incubated with X-gal solution (1 mM 5-bromo-4-chloro-3-indolyl β-D-galactopyranoside, 5 mM K_3_Fe(CN)_6_ and 5 mM K_4_Fe(CN)_6_ in Z-buffer) at 37°C.^57^ The incubation period ranged from 10 min to 24 h, depending on the level of *LacZ* expression, but the corresponding stages of the wild-type and knockout strains were stained for the same length of time. The membranes with stained structures were mounted on slides in 50% glycerol and imaged with a Leica DMLB2 compound microscope.

Cells transformed with the p[*cdl1a*]:*cdl1a-*YFP plasmid were developed on a thin agar. For imaging of developing structures, a small portion of agar was excised and placed in a drop of silicon oil on a coverslip. Confocal microscopic images were obtained with the Leica TCS SP8 platform (Leica Microsystems), and analyzed with Fiji.^58^

#### RNa-seq

Wild-type and *cdl1aˉ* cells were incubated on NN agar until late culminants to mature fruiting bodies had formed, which were dissociated and frozen at −80°C. Total RNA was isolated from three independent experiments and enriched for mRNA using poly-T–linked magnetic beads. Barcoded cDNA libraries were constructed using NEBNext Ultra II Directional RNA Kit for Illumina, following manufacturer’s instructions, and checked for quality using the Agilent TapeStation DNA D1000 HS Kit. The six bar-coded libraries were normalized to 10 nM and combined into one pool, which was sequenced as paired-end 75-bp reads at ∼16 million reads per sample using the Illumina mid-output NextSeq platform (Data S1, sheet 1).

#### Quantitative reverse transcription polymerase chain reaction (qRT-PCR)

Triplicate RNA samples were isolated from 10^7^ cells, each, using the RNeasy Mini Kit (QIAGEN), and DNA contamination was removed using the Turbo DNA-free Kit (Invitrogen). Reverse transcription was performed on 1 μg of RNA with the SensiFAST cDNA synthesis kit (Bioline, UK). The cDNA samples were combined with the oligonucleotide primers listed in Table S2 and PerfeCTa SYBR Green SuperMix (QuantaBio, USA) and amplified using the LightCycler® 96 System (Roche, Germany). The PCR program consisted of 45 cycles, with 30 s at 95°C, 55°C and 72°C each. Gene expression levels were normalized to the mean expression level of the constitutively expressed genes DDB_G0282429 and/or DDB_G0280765^32^ in the same sample.

### Quantification and statistical analysis

#### Quantification and statistical analyses for RNA-seq

The quality of the sequence reads was checked with FastQC (https://www.bioinformatics.babraham.ac.uk/projects/fastqc/). The reads were aligned to the *D. discoideum* genome (GenBank assembly: GCA_000004695.1), counted and quantified with RSEM (–bowtie option)^45^ using the coding-sequence annotation available on dictyBase (https://dictycr.org/). There were three biological replicates each for *cdl1a-* and wild-type. The R package DESeq2^46^ was used to identify differentially expressed genes (DEG) at a false discovery rate (FDR) < 0.05. See Data S1 for Mapped read counts and annotation of DEG.

## Data Availability

• The raw RNA-Seq reads of *cdl1aˉ* and wild-type fruiting bodies have been deposited at the European Nucleotide Archive (study accession number: PRJEB46667) and are publicly available. • This paper does not report original code. • Any additional information required to reanalyze the data reported in this paper is available from the lead contact upon request.

## Supplemental information

Document S1. Figures S1 and S2 and Tables S1 and S2

Data S1. Excel file with the statistical analysis of the RNA-seq experiments comparing transcripts in wild-type and *cdl1a*^*−*^ fruiting bodies, including annotation of differentially expressed genes, related to Figure 2

Data S2. Zip file with the full and trimmed protein and DNA alignments that were used for the phylogenetic trees in Figure 1

Document S2. Article plus supplemental information
