# Supplementary material for: Evolution of a novel cell type in Dictyostelia required gene duplication of a cudA-like transcription factor
Source: Curr Biol. 2022 Jan 24;32(2):428–437.e4. doi: 10.1016/j.cub.2021.11.047 (PMC8808424; doi:10.1016/j.cub.2021.11.047)
Supplement: Document S1. Figures S1 and S2 and Tables S1 and S2 [file mmc1.pdf]

Current Biology, Volume *32*

Supplemental Information

Evolution of a novel cell type in Dictyostelia required gene duplication of a cudA-like transcription factor

Koryu Kin, Zhi-Hui Chen, Gillian Forbes, and Pauline Schaap

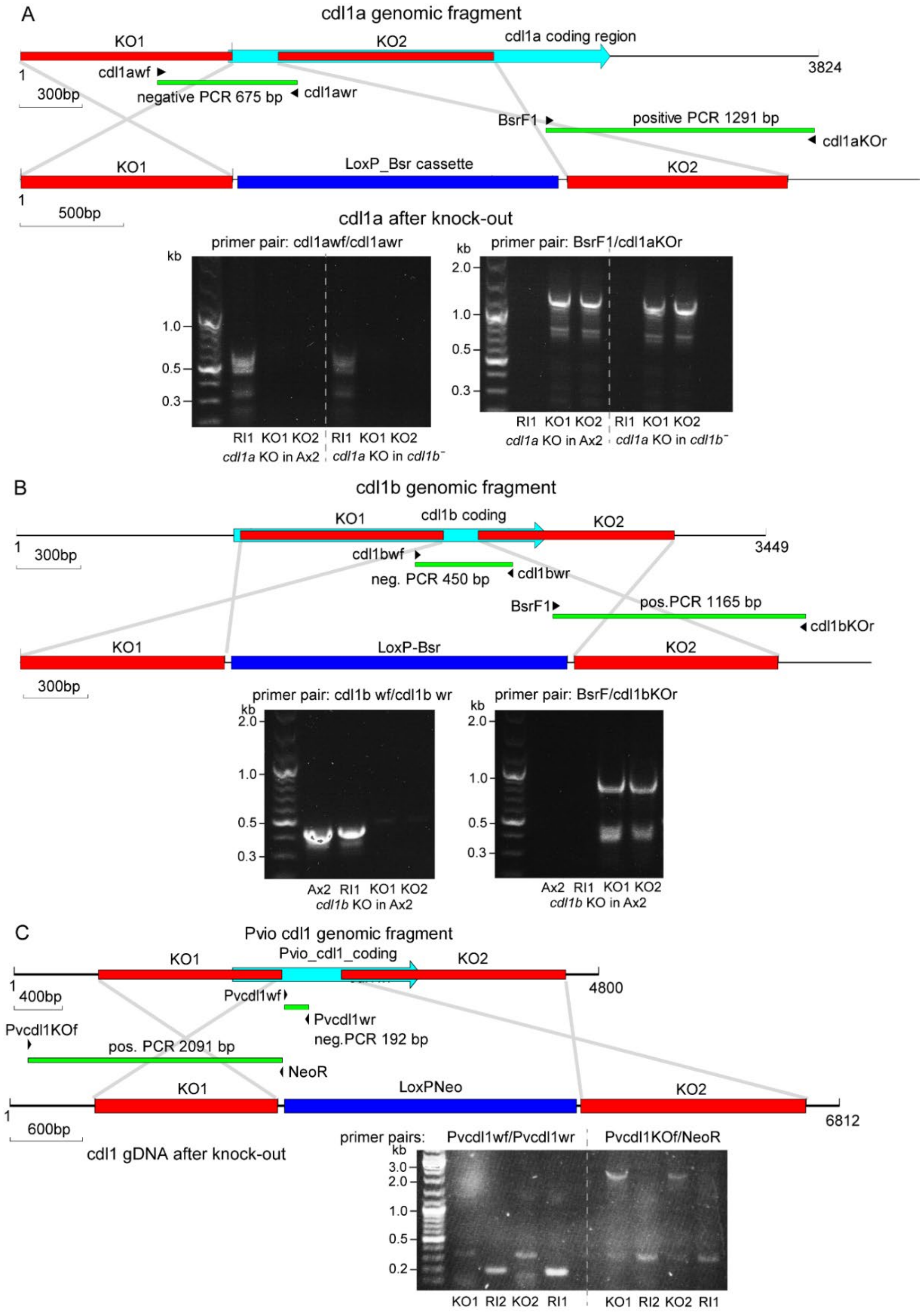

Figure S1. Knock-out constructs and diagnosis, related to Figures 2,4 and 5

Panels A,B and C show the genomic fragments that harbour *D.discoideum cdl1a* (DDB_G0286351) and *cdl1b* (DDB_G0270306), and *P. violaceum cdl1* (Pvio_g1607, Genbank: KAF2077098) respectively, and a schematic of the knockout constructs with the positions of the primers (arrowheads) that were used to diagnose a homologous recombination event in transformed clones. Gel images show the PCR products amplified from genomic DNAs that were isolated from some knockout (KO) or random integrant (RI) clones for each construct.

A. *cdl1a* knockout. Primer pair cdl1awf/cdl1awr (Table S2) amplifies a 0.3 kb fragment from RI, but not from KO gDNAs, while primer pair BsrF1/cdl1aKOr amplifies a 1.3 kb fragment from KO gDNAs only.

B. *cdl1b* knockout. Primer pair cdl1bwf/cdl1bwr amplifies a 0.45 kb fragment from RI gDNAs, while BsrF1/cdl1bKOr amplifies a 1.17 kb fragment from KO gDNAs.

C. *P. violaceum cdl1 knockout.* Primer pair Pvcdl1wf/Pvcdl1wr amplifies a 0.19 kb fragment from RI gDNAs, while cdl1KOf/NeoR amplifies a 2.1 kb fragment from KO gDNAs.

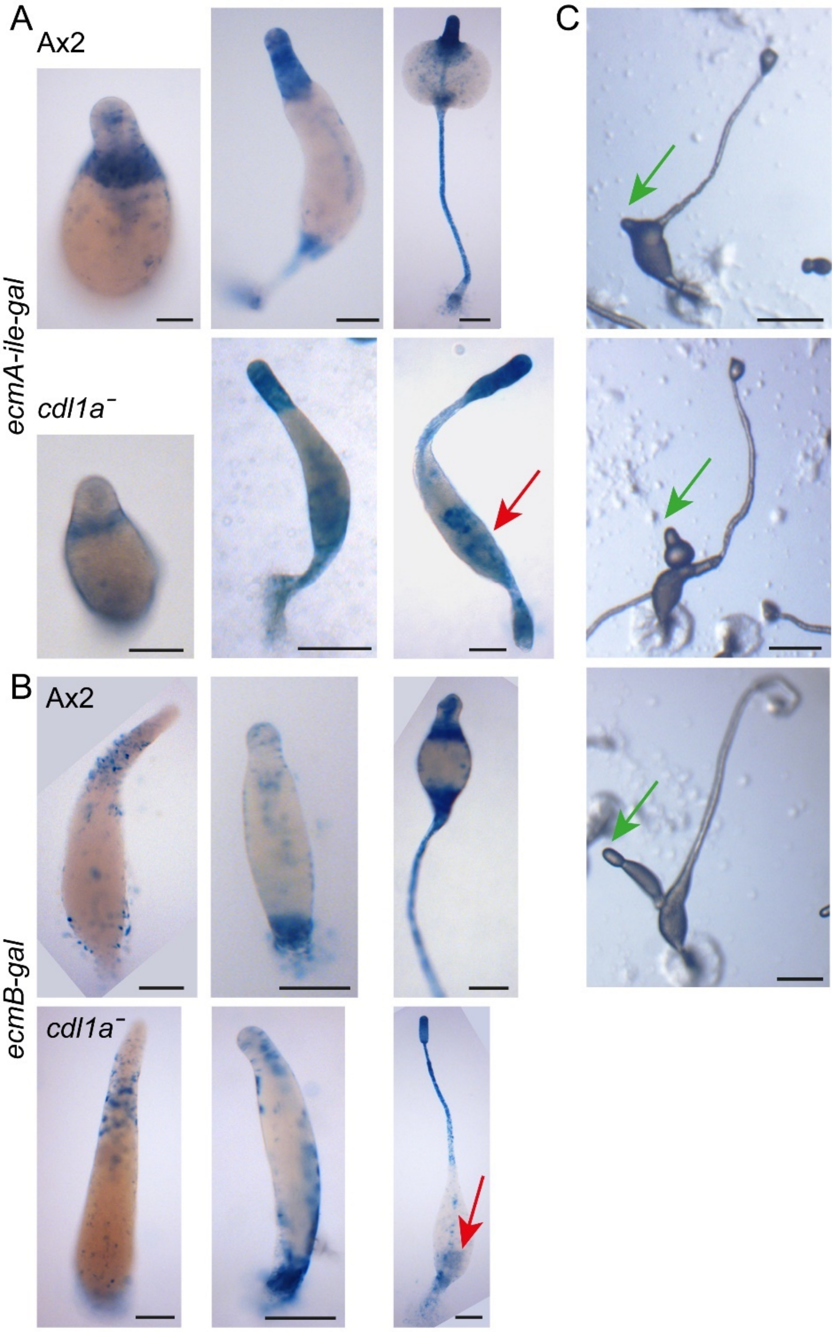

D

	transcripts per million						
Gene	cd1a-1	cd1a-2	cd1a-3	WT-1	WT-2	WT-3	FDR
ecmA	253	289	264	72	165	106	0.658
ecmB	112	243	218	42	147	35	0.638

Figure S2. *ecmA* and *ecmB* expression in *cdl1a*$^{-}$, related to Figure 2
Wild-type Ax2 and *cdl1a*$^{-}$ cells were transformed with pEcmA-ile-gal[S1] or pEcmB-gal constructs[S2] and developed into multicellular structures, which were fixed and stained with X-gal.
A. *EcmA expression patterns*. EcmA-ile-gal positive cells in wild-type (top) and *cdl1a*$^{-}$ structures (bottom) at the early (left), mid (middle), and late culminant stage (right). Bars: 0.5 mm.
B. *EcmB expression patterns*. EcmB-gal positive cells in wild type (top) and *cdl1a*$^{-}$ (bottom) structures at the slug (left), early (middle) and late culminant stage (right). Note the presence of a cluster of cells stained with either ecmA-ile-gal or ecmB-gal in the *cdl1a*$^{-}$ spore mass (red arrows). Bars: 0.5 mm.
C. *Secondary sorogen formation in cdl1a*$^{-}$. After prolonged incubation for more than 2 days, secondary sorogens (green arrows) emerge from the spore mass. The precise timing varies, but the images from early (top) to late (bottom) secondary sorogen formation are shown. Bars: 0.5 mm.

D. Normalized read counts from RNA-Seq data obtained in this study for *ecmA* and *ecmB* in wild-type and *cdl1a*⁻ late culminants (retrieved from Data S1, sheet Mapped_ Reads).

Orthologs in Groups 1, 2 and 3	Cell type specificity	Duplicates in D_discoideum (Group 4)	Cell type specificity	BIPP duplic.	BIPP grps4-3
DFA_06945, PPL_04431, DLA_05575	stalk	DDB_G0270306 (cdl1b)	stalk	1	0.95
		DDB_G0286351 (cdl1a)	cup*/stalk		0.95
DFA_04206, PPL_01589, DLA_07194	spore	DDB_G0281969 (mybE)	cup/spore	duplication not mono phyletic in group 4	
		DDB_G0287637 (mybD)	growth/stalk		
DFA_09741, PPL_02647, DLA_06272	stalk	DDB_G0290765 (jcdA)	growth/stalk/cup	0.81	0.82
		DDB_G0268178 (jcdB)	growth		0.82
DFA_08722, PPL_02053, DLA_01746	growth/spore	DDB_G0289677 (hbx14)	stalk	1	0.74
		DDB_G0277505 (hbx6)	stalk/spore/cup		0.74

Table S1. Possible group 4 specific putative gene duplications in the dictyostelid transcription factors, related to Figure 1. BIPP: Bayesian inference posterior probability of the node combining the gene duplicates and of the node combining both or either of the duplicated gene in group 4 with the group 3 orthologs. Data retrieved from [S3,S4]. *cell types ordered from highest to lowest transcript read counts.

Name	Sequence
cdl1aA	AATggtaccAGTGCGTGTTAGAATGAATGTATTTCTGTGT
cdl1aB	GCACCTTGTTTTGTAATTTCAATTAaagcttGATTA
cdl1aC	AACggatccTCATTCACAACAACACAATAACCAATCGAG
cdl1aD	AATgcggccgcGCAGAAGAAGAGGAAGTAGTAGTGGTAG
cdl1bA	AATggtaccACAAAACAAGTTGCAGTTGCGGTTG
cdl1bB	ATAaagcttGTGTGGAAGTTGAAGAAGAACAGAGTG
cdl1bC	AACggatccTACCAATAGTACACAATTAATTGGG
cdl1bD	AATgcggccgcCAAAACTAAAGGTAGAAATTTAGATAGC
Pvcdl1A	ACggtaccGTTTCACACAAACAACCCCAC
Pvcdl1B	GActcgagCAACGTGTTTGGGTGGATCAG
Pvcdl1C	CCTggatccGGCTCCTCTACTCCATTCTCC
Pvcdl1D	GTtctagaTCCTCCCAGACATTATCCTGTG
cdl1awf	TCAAAATGGTGGTGGTTCAATTTGTGATG
cdl1awr	GAAGCAGAAGAAGAGGAAGTAGTAGTG
BsrF1	GATAAAGCTGACCCGAAAGCTCGGATCTGATATC
cdl1aKOr	TCAGATTCTGGTACGTATTTAAATGCTTCGACAACTC
cdl1aCDSf	AAggatccATGATTTATAATCAAGCTTTAATTGAAATTAC
cdl1aCDSr	AActcgagaAACTATTGGTAATTGTAATAAATCATTTG
cdl1aPRf	gtcgacGTATACTACAAAGAACAAGATG
cdl1aPRr	CAATTAaagcttGATTATAAATCAT
cdl1bwf	CACAAGATAGTCAAAGTAAATGGA
cdl1bwr	GAATTTTGAAAATGACTAGTTGATG
cld1bKOr	TCACTTTTTGACGGTCCACCACCGCAATTAC
Pvcdl1wf	ATGTTGCCACTGTCAATGATG
Pvcdl1wr	TGGCTGTGAATAGAGTTGCTG
Pvcdl1KOf	CAACAAAATACTACAACGACGTG
NeoR	TTAATTAACCCGGGAAGCTTATC

Table S2. Oligonucleotide primers used in this work, Related to STAR Methods
The restriction enzyme sequences that were used for cloning are shown in lower case.

SUPPLEMENTAL REFERENCES

S1. Detterbeck, S., Morandini, P., Wetterauer, B., Bachmair, A., Fischer, K., and MacWilliams, H.K. (1994). The 'prespore-like cells' of Dictyostelium have ceased to express a prespore gene: analysis using short-lived beta-galactosidases as reporters. Development 120, 2847-2855.

S2. Ceccarelli, A., Mahbubani, H., and Williams, J.G. (1991). Positively and negatively acting signals regulating stalk cell and anterior-like cell differentiation in Dictyostelium. Cell 65, 983-989.

S3. Forbes, G., Chen, Z.H., Kin, K., Lawal, H.M., Schilde, C., Yamada, Y., and Schaap, P. (2019). Phylogeny-wide conservation and change in developmental expression, cell-type specificity and functional domains of the transcriptional regulators of social amoebas. BMC Genomics 20, 890. 10.1186/s12864-019-6239-3.

S4. Kin, K., Forbes, G., Cassidy, A., and Schaap, P. (2018). Cell-type specific RNA-Seq reveals novel roles and regulatory programs for terminally differentiated Dictyostelium cells. BMC Genomics 19, 764. 10.1186/s12864-018-5146-3.
